# Brain Delivery of Thyrotropin-Releasing Hormone via a Novel Prodrug Approach

**DOI:** 10.3390/pharmaceutics11070349

**Published:** 2019-07-18

**Authors:** Katalin Prokai-Tatrai, Daniel L. De La Cruz, Vien Nguyen, Benjamin P. Ross, Istvan Toth, Laszlo Prokai

**Affiliations:** 1 Department of Pharmacology and Neuroscience, University of North Texas Health Science Center, Fort Worth, TX 76107, USA; 2 School of Pharmacy, The University of Queensland, Brisbane, QLD 4072, Australia; 3 School of Chemistry & Molecular Biosciences, The University of Queensland, St Lucia, QLD 4072, Australia

**Keywords:** TRH, brain-targeting prodrug design, prolyl oligopeptides (POP), lipoamino acid, CNS-drug delivery

## Abstract

Using thyrotropin-releasing hormone (TRH) as a model, we explored whether synergistic combination of lipoamino acid(s) and a linker cleaved by prolyl oligopeptidase (POP) can be used as a promoiety for prodrug design for the preferential brain delivery of the peptide. A representative prodrug based on this design principle was synthesized, and its membrane affinity and in vitro metabolic stability, with or without the presence of a POP inhibitor, were studied. The in vivo formation of TRH from the prodrug construct was probed by utilizing the antidepressant effect of the peptide, as well as its ability to increase acetylcholine (ACh) synthesis and release. We found that the prototype prodrug showed excellent membrane affinity and greatly increased metabolic stability in mouse blood and brain homogenate compared to the parent peptide, yet a POP inhibitor completely prevented prodrug metabolism in brain homogenate. In vivo, administration of the prodrug triggered antidepressant-like effect, and microdialysis sampling showed greatly increased ACh release that was also antagonized upon a POP inhibitor treatment. Altogether, the obtained promising exploratory data warrant further investigations on the utility of the prodrug approach introduced here for brain-enhanced delivery of small peptides with neurotherapeutic potential.

## 1. Introduction

**Figure 1:**
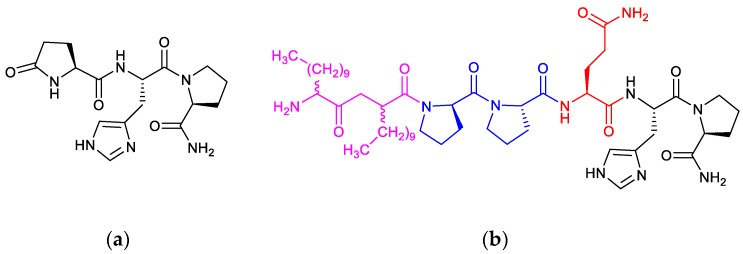
Chemical structure of (**a**) Thyrotropin-releasing hormone (TRH) and (**b**) its C12-C12-Pro-Pro-Gln-His-Pro-NH_2_ prodrug (**1**) extended from the N-terminus of Gln-His-Pro-NH_2_ (**2**) with a pair of 2-aminododecanoyl (C12) residues as lipoamino acids (LAAs) (magenta) and a dipeptidyl (Pro-Pro) prolyl oligopeptidase (POP)-sensitive linker (blue).

Thyrotropin-releasing hormone (TRH, pGlu-His-Pro-NH_2_) shown in Figure 1a, is an excellent representative of small neuropeptides with unfulfilled neurotherapeutic potential for the treatment of neurological and psychological disorders due to its inherent ability to alter brain chemistry and, subsequently, behavior and physiology [1,2,3,4,5]. This peptide is abundant in the central nervous system (CNS) and believed to act through two known (TRH-R1 and TRH-R2) receptors [2]. Notable neuropharmacological effects of TRH include homeostatic modulation and, thus, regulation of arousal and seizure activity, as well as antidepressant and neuroprotective properties [1,6]. TRH’s robust modulation of acetylcholine (ACh) synthesis and release may also benefit diseases exhibiting symptoms of decreased cognitive function such as Alzheimer’s disease [3]. As with peptides in general, and especially in the context of non-invasive brain delivery for potential neurotherapy, TRH suffers from numerous shortcomings including inadequate metabolic stability in the periphery [6,7]. To elicit desired CNS-effects, large doses are needed, which in turn trigger unwanted neuroendocrine side effects. Two main directions have been explored to harness the beneficial effects of the peptide in the CNS, while overcoming limitations of its direct delivery from the circulation. One approach has focused on creating analogs with diminished neuroendocrine activity, as well as increased metabolic stability and oral bioavailability [8,9,10]. Among these analogs, taltirelin has been approved in Japan for human therapy against spinocerebellar ataxia [10].

Another strategy has considered transient modifications of the peptide’s insufficient physicochemical and metabolic properties for transport across the blood-brain barrier (BBB) by prodrug designs [7,11,12,13,14,15]. Prodrugs are inert until they are metabolized in vivo into the active (parent) drug. For site-enhanced delivery, metabolizing enzymes preferentially expressed at the site of action are desired. Previously, we studied 1,4-dihydropyridine (DHP)-based prodrugs which provided enhanced brain delivery of the parent agents compared to those of simple prodrugs [16,17,18]. However, several shortcomings associated with this approach prompted us to seek alternative brain-targeting prodrug approaches. Specifically, tractability issues owing to the intrinsic sensitivities of DHPs to oxidation, even at ambient conditions in the air [19], and their ability to undergo nucleophilic addition to the 5,6-double bond (such as water addition) even under mildly acidic conditions could easily destroy the prodrug construct. Additionally, there have been concerns about the effect of pyridinium salts in the brain, as well as the peripheral impacts of DHP- and pyridinium-based agents [20,21] abolishing altogether the realistic translational value of this approach.

These serious limitations were unavoidable even when the DHP promoiety was combined with specific enzyme-sensitive linkers attached to the amino-terminus of small peptides for their preferential release from the prodrug construct in the brain [12,22,23]. Such enzyme-sensitive (dipeptidyl or single amino acid) linkers were based on the inherent feature of prolyl oligopeptidase (POP, a.k.a. post-proline cleaving enzyme) [24]. POP selectively hydrolyzes a peptide bond at the carboxyl side of internal proline in peptides of up to 30 amino acid residues long due to recognition of the unique conformational constraint that this cyclic amino acid residue brings into an oligopeptide chain. Since the highest activity of POP was found in the brain and owing to its uneven distribution within this organ [25], POP-activated prodrugs also may be useful to target selected regions of the brain.

As lipophilicity is a major governing factor for diffusion through biological membranes including the BBB [26], providing adequate lipophilicity of prodrugs has been a well-recognized strategy in most prodrug designs aiming at drug delivery from the circulation. Lipoamino acids (LAAs) have been introduced to augment lipophilicity and introduce amphiphilicity into the structures to facilitate interactions with cell membranes [27,28]. Side-chain manipulation of LAA residues also allows for tailoring to the desired physicochemical properties to enhance organ uptake. Utilization of LAA alone for creating peptide prodrugs targeting the brain would, however, not result in useful prodrugs, when LAA is attached directly to the N-terminus because of the general stability of amides against enzymatic degradation in the brain [7,17,24]. Therefore, LAA transport moieties need to be combined with appropriate peptide sequences recognized by specific brain peptidases, such as POP [24], allowing for their preferential removal therein.

**Scheme 1:**
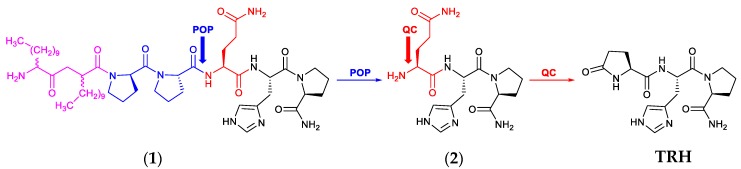
Schematic illustration of the bioactivation of the prototype prodrug (**1**) to TRH in the brain. The LAA moiety (magenta) with the POP-sensitive linker (blue) is removed by POP liberating Gln-His-Pro-NH_2_ (**2**) whose amino-terminal residue (red) is converted by QC to pGlu leading to the formation of TRH in the brain.

Accordingly, in the present study we explored whether a synergistic combination of the LAA-based transport moiety and a POP-sensitive linker could be exploited for prodrug design to deliver TRH into the brain. Since TRH itself does not possess useful functional groups for prodrug synthesis, we replaced its N-terminus with Gln. The latter is efficiently converted to pGlu by glutaminyl cyclase (QC) [7,22] thereby resulting in the formation of TRH from Gln-His-Pro-NH_2_ (**2**) in the brain, as shown in Scheme 1. As a prototype of this design principle, we have synthesized C12-C12-Pro-Pro-Gln-His-Pro-NH_2_ (**1**) prodrug that carries two 2-aminododecanoyl (C12) residues as LAA, and a diprolyl POP-sensitive linker attached to the N-terminus of (**2**) (Figure 1b, Scheme 1). In a comparative fashion using TRH as reference, we have assessed this prodrug’s affinity to biological membranes as a predictor for BBB permeability using immobilized artificial membrane chromatography (IAMC) [29] and evaluated its in vitro stability in relevant biological matrices. To confirm the crucial role of POP in the design, in vitro metabolic stability studies were also conducted in the presence of a POP-inhibitor (KYP-2047) [30]. As initial in vivo surveys of the utility of the prodrug approach introduced here, we utilized the Porsolt swim test (PST) in mice and in vivo microdialysis sampling in rats to follow the formation of TRH from (**1**) relying on well-known neuropharmacodynamic (antidepressant) and neurochemical (modulation of ACh synthesis and release) effects of the parent TRH, respectively [6].

## 2. Materials and Methods

### 2.1. Materials

All reagents and solvents were obtained from commercial sources.

### 2.2. Instruments

Preparative gradient reversed-phase (RP) high-performance liquid chromatography (HPLC) was done on a Waters (Milford, MA, USA) system consisting of a Series 600 controller, a 600 F pump and a 490 E ultraviolet (UV) spectrophotometric detector operated at 230 nm. Analytical gradient RP-HPLC was performed on a Shimadzu (Kyoto, Japan) HPLC system using UV detection at 214 nm. High-resolution mass spectrum (HR-MS) was acquired on a Thermo Scientific (San Jose, CA, USA) LTQ Velos Orbitrap Pro instrument operating in positive electrospray ionization (ESI) mode and using 50:50 *v*/*v* methanol:water containing 0.1% *v*/*v* formic acid as solvent. IAMC-MS analyses in positive-ion ESI mode were done on a ThermoQuest LCQ-Deca ion trap mass spectrometer (Thermo Fisher Scientific; Waltham, MA, USA) coupled with a Surveyor MS LC system (Thermo Fisher Scientific). LC–ESI-MS for in vitro studies was done on a TSQ Quantum Ultra triple-quadrupole mass spectrometer coupled with a Surveyor HPLC system (Thermo Fisher Scientific). Another Thermo Finnigan TSQ Quantum Ultra triple quadrupole tandem MS connected to a Vanquish Flex LC (Thermo Fisher Scientific) was utilized for the analyses of in vivo microdialysis samples to establish extracellular ACh levels.

### 2.3. Animals

Male CD-1 mice (43 ± 6 g) and male Sprague-Dawley rats (275 ± 6 g body weight) were obtained from Envigo Corporation (Indianapolis, IN, USA). They were housed two rats or five mice per cage in the DLAM facility at the University of North Texas Health Science Center on a common 12-h/12-h light dark cycle with free access to food and water. All procedures were reviewed and approved by the institutional animal care and use committee before the initiation of the studies (approval numbers 2014/15-22-A04, 2014/15-22-A05, 2018-0004 and 2018-0006). Each animal was used only once for in vivo studies.

### 2.4. Synthesis

Prodrug (**1**), as the prototype for the design principle introduced here, was prepared by manual solid-phase peptide chemistry using 9-fluorenylmethyloxycarbonyl (Fmoc-)protected amino acids on Rink amide 4-methylbenzhydrylamine (MBHA) resin (100–200 mesh, 0.73 mmol/g resin loading) in a customary manner [31] utilizing 2-(1H-benzotriazol-1-yl)-1,1,3,3-tetramethyl-uronium hexafluorophosphate/*N*,*N*-diisopropylethylamine (HBTU/DIPEA) activation. Once Pro-Pro-Gln-His-Pro-NH_2_ was assembled on the solid support, it was further elongated by two consecutive couplings with 2-[(4,4-Dimethyl-2,6-dioxocyclohex-1-ylidene)ethylamino]-d,l-dodecanoic acid (N-Dde-C12-OH) that was prepared as reported before [32]. The Dde protecting group was removed with 2% (*w*/*v*) hydrazine hydrate. The crude product was cleaved from the resin with a solution containing 95% (*v*/*v*) TFA, 2.5% (*v*/*v*) water and 2.5% (*v*/*v*) triisopropylsilane (20 mL/g resin) for 2 h. The product was purified by preparative gradient RP-HPLC using a Vydac (Grace Davison Discovery Sciences, Rowville, Australia) 250 × 22 mm i.d., packing particle size (*d*_p_) = 10 µm octadecylsilica (C18) column. The purity of the prodrug was determined by analytical gradient RP-HPLC using two different chromatographic conditions: System 1) Agilent (Palo Alto, CA, USA) Zorbax SB-C18 column (150 × 4.6 mm i.d., *d*_p_ = 3.5 µm) fitted with a guard cartridge (C18 SecurityGuard, 4 × 3.0 mm i.d.; Phenomenex, Torrance, CA, USA); System-2) Vydac 218TP54 C18 column (250 × 4.6 mm i.d., *d*_p_ = 5 µm) fitted with a guard cartridge (Phenomenex C18 SecurityGuard, 4 × 3.0 mm i.d.). The mobile phase was solvent A (0.1% *v*/*v* TFA in water) and solvent B (0.1% *v*/*v* TFA in 90% *v*/*v* acetonitrile/10% *v*/*v* water) for both systems. Purity was calculated as the percent area of integrated peaks in the analytical RP-HPLC chromatogram. Estimated purities were 97% and 98% using Systems 1 and 2, respectively. HR-MS: calc. *m/z* for [M + H]^+^ 968.6655 (C_50_H_86_N_11_O_8_), found 968.6652; calc. *m/z* for [M + 2H]^2+^ 484.8364 (C_50_H_87_N_11_O_8_), found 484.8354.

### 2.5. Membrane Affinity Studies

An IAM.PC.DD2 column (3 cm × 4.6 mm i.d., *d*_p_ = 12 µm, from Regis Technologies Inc.; Morton Grove, IL, USA) was used for IAMC studies according to the method of Valko et al. [29]. Nicotine, caffeine, hydrocortisone, progesterone, procaine, and propranolol were chosen as reference standards. Gradient elution at flow rate of 1.0 mL/min was used. The binary eluent consisted of (A) 50 mM aqueous ammonium acetate buffered at various pHs (7.0, 6.1, and 5.4 set with the addition of 1 M acetic acid) and (B) acetonitrile, and the gradient program was linear from 0% to 80% B in 15 min. At each designated pH of solvent A, the reference compounds’ gradient retention times were determined to establish a linear relationship, from which the retention time of an analyte could be used to determine its IAMC chromatographic hydrophobicity index (CHI_IAM_) value.

### 2.6. In Vitro Metabolic Stability Studies

Metabolic stability studies were carried out as reported before [14,33] using freshly made brain homogenate (20% *w*/*v*) and plasma at 37 °C, in triplicate. For obtaining brain homogenates, a Bead Bug microtube homogenizer (Benchmark Scientific, Edison, NJ, USA) was used. For obtaining plasma, blood was collected in BD Vacutainer^®^ Sodium Heparin tubes (Becton, Dickinson and Co., Franklin Lakes, NJ, USA) followed by centrifugation. Each biological matrix was spiked with compound (**1**) or TRH to create 10 µM solutions. KYP-2047, a POP inhibitor, was used in selected experiments at 50 µM concentration [30]. Sample aliquots (100 µL) of TRH-containing matrices were removed after 2, 4, 6, 8, 10, 15, and 30 min, while sample aliquots (100 µL) of prodrug-containing matrices were obtained at 5, 10, 20, 30, 40, 60, 90, 120, and 210 min. Sample aliquots were added to 200 µL of acetic acid solution (20% *v*/*v*) in acetonitrile, thoroughly vortexed and, then, centrifuged before LC–MS/MS analysis. For LC separation, a Kinetex-C8 column (10 cm × 4.6 mm i.d., *d*_p_ = 2.6 µm; Phenomenex; Torrance, CA, USA) with the eluent mixed from (A) H_2_O with 0.1% (*v*/*v*) formic acid and (B) acetonitrile with 0.1% (*v*/*v*) formic acid, flow rate of 400 µL/min, and gradient elution by a 10-min linear program from 0% B to 90% B were employed. Half-life (*t*_1/2_) values were calculated by assuming pseudo-first-order degradation.

### 2.7. Neuropharmacodynamic Assesment: PST

For the assessment of antidepressant-like activity after subcutaneous (s.c.) administration of (**1**), our validated and previously published PST protocol was used [16,34]. TRH (3 µmol/kg body weight) was the positive control. Eight mice per treatment group were tested. The prodrug was also administered at 3 µmol/kg body weight dose. Thirty min after drug exposure, the behavioral test began. It was conducted for 6 min, and immobility time was recorded 2 min after the initiation of the study. The behavior of the animals was recorded and three trained observers, blinded to the treatment protocol, independently analyzed the videos to establish immobility times.

### 2.8. Neurochemical Assestment: ACh Release Using In Vivo Microdialysis

To assess TRH’s modulation of ACh after its in vivo liberation from the prodrug, a pilot study utilizing microdialysis sampling of the extracellular space of rat frontal cortex was done according to our previously published protocol [16,35] with modifications. These modifications included ESI coupled with tandem mass spectrometry (MS/MS) instead of amperometry to measure ACh concentrations [36] after HPLC separation of the analyte, which also allowed us to avoid the use of the acetylcholinesterase inhibitor neostigmine in our experiment as an auxiliary (and potentially confounding) agent. Briefly, a cerebral guide cannula (CMA/Microdialysis, Inc.; Acton, MA, USA) was inserted into the frontal cortex; before the actual microdialysis probe (CMA/12, CMA/Microdialysis, Inc., with a 4-mm long polycarbonate membrane having 20,000 Da molecular weight cut-off) was inserted about one week after placement of the guide cannula. Artificial cerebral spinal fluid (aCSF) was used as the perfusion medium at a perfusion rate of 2 μL/min. The perfused prodrug and TRH concentrations were 3 mM in aCSF. The brain-permeable KYP-2047 as a POP inhibitor [30] was administered intraperitonially (i.p., 50 µmol/kg) 40 min after the perfusion of the prodrug started. Samples (20-min fractions) were collected in a refrigerated fraction collector (Bioanalytical Systems, Inc., West Lafayette, IN, USA). HPLC separation was done on a mixed-mode Scherzo SS-C18 column (50 × 2 mm i.d., *d*_p_ = 3 µm; Imtakt Corp., Kyoto, Japan) at a flow rate of 400 µL/min. ACh was eluted using 0.5% *v*/*v* formic acid in water as solvent A, and 100 mM aqueous ammonium acetate/acetonitrile (65/35, *v*/*v*) as solvent B with linear gradient from 0 to 60% B in 6 min. Quantification was based on isotope-dilution ESI-MS/MS using d_4_-ACh as internal standard [36]. Baseline fractions were used as each animal’s own control. Steady-state ACh levels after TRH and prodrug perfusions were expressed as percentiles, respectively, taking baseline ACh levels before perfusions as 100%.

### 2.9. Statistical Analysis

Statistical evaluations were done by one-way analysis of variance (ANOVA). Two-group comparisons employed post hoc Tukey tests, and *p* < 0.05 was considered statistically significant.

## 3. Results and Discussion

The design principle to assemble prodrugs to deliver small peptides from the circulation into the brain was based on the expectation that extension of a small peptide on its amino-terminus with LAA + POP-sensitive linker as a “promoiety” (Scheme 1) would produce prodrug conjugates capable of preferentially releasing the parent peptide at the site of action owing to brain-enhanced expression of POP compared to the rest of the body [25]. In this exploratory study to test our hypothesis, we have selected TRH as a model peptide having tremendous neurotherapeutic potential [1,2,3,4,5,6]. To gain a suitable functional group for prodrug synthesis, we used (**2**) that carries Gln instead of pGlu at the amino-terminus [7,22]. According to Scheme 1, we anticipated that TRH would preferentially be liberated from such type of prodrug construct, represented here by (**1**) and shown in Figure 1b, within the brain. This approach would also overcome metabolic instability issues of the parent peptide [1,6].

### 3.1. Synthesis and IAMC Studies

We have assembled C12-C12-Pro-Pro-Gln-His-Pro-NH_2_ (**1**) as the prototype of the design principle having prolyl-prolyl (Pro-Pro) as a POP-sensitive linker extended on its N-terminus with two C12 (2-amino-d,l-dodecanoyl) residues as LAA (Figure 1b). The synthesis was done via traditional manual solid-phase and Fmoc-based peptide chemistry using HBTU/DIPEA activation [31]. N-Dde-protected racemic 2-aminododecanoic acid (C12-OH) [32] was used to introduce the LAA moiety, and the Dde protecting group was removed with hydrazine after coupling. As far as the LAA selection was concerned, we reasoned that a sufficiently long-chain LAA was needed to provide adequate lipophilicity for the prodrug construct for favorable BBB permeation. Indeed, the calculated [37] logarithm of the *n*-octanol–water partition coefficient (clogP) for the prodrug (**1**) was 1.85 (Table 1), while in contrast TRH’s clogP was −3.50, indicating the overwhelmingly hydrophilic character of the latter. We are aware of size limitation of molecular transport across the BBB, but we also wanted to prove through the introduction of two LAAs that the mechanism of LAA-facilitated increase of peptide transport [27,28] would not be limited by the strict, approximately 400-Da molecular weight BBB exclusion considered for non-biologics [26]. Initial selection of the POP-sensitive Pro-Pro linker was based on our earlier observations where this linker provided the most efficacious prodrugs [7,23].

The prototype prodrug’s membrane affinity was estimated in a comparative fashion with that of TRH by IAMC, where the chromatographic column mimicked biological membranes [38,39]. Using the approach introduced by Valko et al. [29], we first determined the chromatographic hydrophobicity index from the IAMC retention times (CHI_IAMC_) for the prodrug and TRH, respectively. Table 1 shows the average CHI_IAM_ values when retention times were measured at three different pH values (7.0, 6.1, and 5.4). Expectedly, the prodrug was predicted to have a significantly increased affinity to biological membranes, such as the BBB, compared to the highly hydrophilic parent peptide. The clogP values also showed the expected trend in lipophilicity; moreover, the clogP of the prodrug (1.85) was in the favorable logP range for transport across the BBB from the circulation [26], while only one C12 as LAA in the structure would not have provided sufficient increase in lipophilicity (with corresponding clogP of −1.41).

### 3.2. In Vitro Metabolic Stability Studies

Owing to the metabolic instability of TRH in the circulation and elsewhere in the body [1,6,7], one purpose of the prodrug design should be the improvement of metabolic stability—especially in the circulation to ensure adequate brain uptake in the context of utilizing TRH as a neuropeptide. We anticipated that the prototype prodrug (**1**) would exhibit a significantly increased metabolic stability in plasma compared to that of TRH, while it would be less stable in brain homogenate to ensure TRH’s release in the organ. As shown in Table 2, the prodrug was quite stable in plasma with t_1/2_ of 100 ± 7 min, while t_1/2_ was significantly shorter (47 ± 6 min) in brain homogenate; yet this is a considerable increase compared to TRH’s biological *t*_1/2_ of about 7 min in plasma and less than 5 min in CD-1 mouse brain homogenate.

Previously published TRH’s *t*_1/2_ values were determined in rat tissues [8]; nevertheless, our data did not statistically differ from those obtained in rats. We selected CD-1 mice-derived tissues for this study to forecast the in vivo potential of the prodrug to trigger the well-known antidepressant-like effect of TRH [1,2]. The significantly higher metabolic stability of the prodrug in plasma should be advantageous not only in terms of brain uptake of the intact prodrug from the circulation, but, also very importantly, one may expect significantly reduced TRH exposure thereby decreasing unwanted endocrine side-effect in the periphery following systemic administration of (**1**).

When KYP-2047, a potent POP inhibitor [30], was added to the brain homogenate together with the prodrug, the latter exhibited remarkable stability (*t*_1/2_ > 24 h), in contrast with *t*_1/2_ of 47 ± 6 min without the enzyme inhibitor. This finding highlights the crucial role of POP in our prodrug’s bioactivation in the brain and confirms the reported high POP activity therein [25]. Surprisingly, the prodrug’s half-life did not change in plasma when the POP inhibitor was added—indicating that POP activity in the CD-1 male mouse plasma may not be prevalent, and peptidases other than POP are responsible for the metabolism of the prodrug in the circulation. Altogether, in vitro metabolic stability studies revealed that the prodrug is quite stable in plasma (*t*_1/2_ = 100 ± 7 min), potentially predicting a diminished endocrine exposure to the periphery upon its administration. In the brain, POP is indeed the metabolizing enzyme as proposed in Scheme 1, because the presence of KYP-2047 blocked the removal of the (C12)_2_-(Pro)_2_-promoiety (Table 2), which thereby prevented the release of Gln-His-Pro-NH_2_ (**2**) and, thus, the subsequent formation of TRH.

### 3.3. In Vivo Studies

We used two typical CNS effects of TRH for the initial in vivo evaluation of our prodrug design; we have focused on well-known neuropharmacodynamic and neurochemical effects of the parent peptide. Specifically, the antidepressant-like activity modelled by the PST [1,6], as well as the peptide’s ability to increase ACh formation and extracellular release [6] were probed upon prodrug administration.

#### 3.3.1. Antidepressant-Like Effect

**Figure 2:**
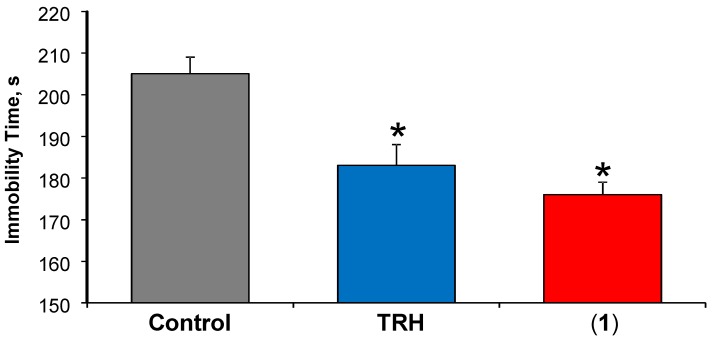
The antidepressant-like effect of TRH after subcutaneous (s.c.) administration of (**1**) at the dose of 3 µmol/kg body weight. Equimolar dose of TRH was used as a positive control. Data is expressed as average ± standard error, *n* = 8. Asterisks indicate statistically significant differences (*p* < 0.05) from the vehicle control group using one-way ANOVA followed by post hoc Tukey test.

In the PST, the immobility represents a “depressive mood” and, when mice receive a test agent with antidepressant-like effect, the immobility time is reduced compared to that of the control group, as mice spend more time swimming in order to escape. TRH has been shown by us [6,13,16,34] and others [40] to exhibit such a central effect. As shown in Figure 2, a statistically significant decrease in the immobility time was established compared to mice receiving vehicle only (immobility time of 205 ± 4 s), when the prodrug was injected to the animals (immobility time of 176 ± 3 s). However, the observed antidepressant-like effect of TRH released from the prodrug was statistically not different from that of direct administration of equimolar TRH (immobility time of 183 ± 5 s).

While the prodrug did not perform significantly better than the direct administration of TRH in this paradigm under the experimental conditions employed [16,34] we feel that these exploratory results are encouraging especially in the context of potential preclinical therapeutic advantage considering the excellent in vitro stability of the prodrug in plasma (Table 2). Therefore, we anticipate that prodrug administration will be devoid of significant TRH exposure to the periphery in follow up studies. Additionally, we propose that the flexibility of the design would also allow for easy modifications of the promoiety (LAA + POP-sensitive linker) to optimize the release of TRH from other, alternative prodrug constructs built on the design principle introduced here.

#### 3.3.2. ACh Release

**Figure 3:**
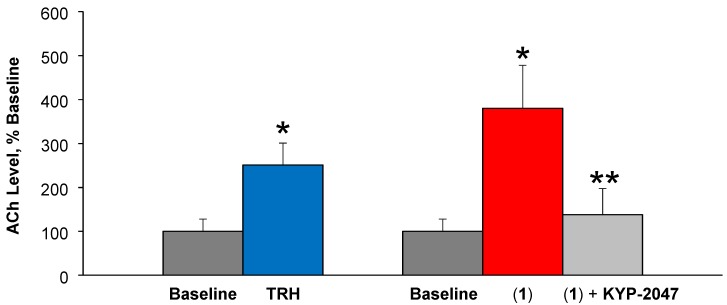
Percent acetylcholine (Ach) increase compared to steady-state baseline ACh concentration measured in samples collected before perfusion of the test agents taken as 100%. In the rat frontal cortex, prodrug (**1**) perfusion elicited an about 4-fold increase, while TRH as a positive control produced a 2.5-fold increase in ACh. * Indicates statistically significant difference (*p* < 0.05) from baseline control. ** Indicates statistically significant difference (*p* < 0.05) from prodrug perfusion, but no difference from the baseline.

Another exploratory evaluation of the prodrug design employed in vivo microdialysis sampling from the extracellular space of the rat cortex for monitoring ACh release by TRH [34,35], when the prodrug was perfused with or without the presence of the brain-permeable POP inhibitor [30]. TRH is well-known for its ability to increase ACh production, and this cholinergic mechanism has been implicated in many CNS effects of the peptide [1,6]. As shown in Figure 3, perfusion of (**1**) produced an about 4-fold increase in steady-state ACh level compared to baseline, while TRH as positive control brought about a 2.5-fold increase, in agreement with our previous findings [35]. Here, the steady-state ACh levels upon aCSF only perfusions were considered 100% (baseline), and steady-state ACh levels reached after subsequent perfusion with a test agent were expressed as percentages compared to this baseline. The significant increase in ACh upon prodrug perfusion have confirmed the ability of POP to release Gln-His-Pro-NH_2_ (**2**) that is then converted to TRH by QC according to Scheme 1. A completely different outcome was observed when animals received the brain-permeable POP inhibitor 40 min after the start of prodrug perfusion; i.e., KYP-2047 completely prevented TRH formation from (**1**) and, thus, no increase in extracellular ACh was observed providing thereby important proof-of-concept results regarding our prodrug approach. Notably, the presence of TRH could also be detected in applicable perfusates upon analyzing them by LC–ESI-MS/MS. Due to the rapid metabolic degradation of TRH (Table 2), we have not been able to capture the intact peptide in brain homogenate in our in vitro studies, when prodrug metabolism in this matrix was monitored.

## 4. Conclusions

In summary, in this exploratory study we have evaluated a novel prodrug approach for peptide delivery into the brain. We selected TRH with unfulfilled neurotherapeutic potential as a model peptide for exploring whether a synergistic combination of LAA and a POP–sensitive linker as a promoiety (Scheme 1) would produce useful prodrugs in this regard. In a comparative fashion using TRH as a reference, we have evaluated our prototype prodrug C12-C12-Pro-Pro-Gln-His-Pro-NH_2_ (**1**) first for membrane affinity as a predictor for BBB uptake from the circulation (Table 1) and for its in vitro metabolic stability in rodent brain and blood (Table 2). Our IAMC study showed that the prodrug had greatly improved membrane affinity compared to the highly hydrophilic parent peptide, and clogP of 1.85 also predicted a favorable BBB transport from the circulation. In vitro studies also revealed a sufficient metabolic stability of (**1**) in plasma predicting a potentially diminished TRH exposure of the periphery upon systemic prodrug administration. In contrast, this prototype prodrug was much less stable in brain homogenate; moreover, it resisted degradation in the presence of a POP inhibitor. Two in vivo experiments using animal models of typical central TRH effects (Figure 2 and Figure 3) have also resulted in promising results and, therefore, warrant further investigations on the utility of the prodrug approach introduced here for the brain-enhanced delivery of small peptides with neurotherapeutic potential.

## Figures and Tables

**Table 1:** Prediction of blood-brain barrier (BBB) permeability of prodrug (**1**) versus TRH. CHI_IAM_ was determined according to Valko et al. [29], while clogP was calculated using an atom-typing additive method [37].

Compound	CHI_IAM_^1^	clogP ^2^
TRH	−11.2 ± 3.2	−3.50
(**1**)	66.0 ± 10.3	1.85

^1^ Average ± standard deviation of measurements at three pH values; ^2^ SCIGRESS software. (version FJ 2.8.1; Fujitsu–FQS, Poland), built-in application.

**Table 2:** In vitro stability in male CD-1 mouse plasma and brain homogenate. Test compounds were used at 10 µM concentration, while KYP-2047 was used at 50 µM. Data represent averages, n = 3, indicated errors are standard deviations.

Test Compound	*t*_1/2_ in Plasma	*t*_1/2_ in 20% (*w*/*v*) Brain Homogenate
TRH	7 ± 4 min	4 ± 1 min
(**1**)	100 ± 7 min	47 ± 6 min
(**1**) + KYP-2047	115 ± 13 min	>24 h
